# 3-hydroxy butyrate dehydrogenase 2 deficiency aggravates systemic lupus erythematosus progression in a mouse model by promoting CD40 ligand demethylation

**DOI:** 10.1080/21655979.2022.2025694

**Published:** 2022-01-09

**Authors:** Bo Yang, Shihao Hou, Jingjing Zhao, Yepeng Li

**Affiliations:** a Department of Oncology, The Affiliated Hospital of Youjiang Medical University for Nationalities, Baise, P.R. China; b School of Clinical Medicine, Graduate School of Youjiang Medical University for Nationalities, Baise, P.R. China

**Keywords:** Systemic lupus erythematosus, CD4+ T cells, CD40L, 3-hydroxy butyrate dehydrogenase 2, demethylation

## Abstract

The implications of the CD40-CD40 ligand (CD40L) signaling pathway in systemic lupus erythematosus (SLE) were well documented, due to its important role among immune cells. Previous research found that 3-hydroxy butyrate dehydrogenase 2 (BDH2), a modulator of intracellular iron homeostasis and iron transportation promoted the pathogenic process of SLE by regulating the demethylation of *cd70, cd11a*, and *cd40l* genes among CD4 + T cells. The purpose of this study was to explore the role of BDH2 in oxidative damage-induced SLE. First, CD4 + T cells treated with H_2_O_2_ were injected into the tail vein of mice to establish a lupus model. CD40L knockdown significantly decreased CD40L expression on CD4 + T cells in the spleen of SLE mice. Compared with SLE model mice, the levels of serum anti-dsDNA antibody and urinary protein in the CD40L interference group were significantly decreased. CD40L knockdown alleviated the immune complex glomerulonephritis in syngeneic SLE mice. Moreover, the levels of IFN-γ and IL-2 were decreased. However, IL-4 and IL-10 levels were significantly upregulated in the serum of CD40L knockdown SLE mice, compared with SLE model mice. Accordingly, CD40L knockdown reduced Th1/Th2 percentage in SLE mice. Inhibiting the expression of BDH2 of CD4 + T cells promoted the demethylation of CD40L, while it inhibited cell proliferation, elevated oxidative stress through increased expression of CD40L, and thus, promoted the progress of SLE. Our results demonstrate that BDH2 aggravates the pathologic progression of SLE in mice, by increasing the demethylation level of CD40L among CD4 + T cells.

## Introduction

Lupus nephritis (LN) is one of the most severe complications of the Systemic Lupus Erythematosus (SLE), which is recognized as a chronic and prototypical antibody-mediated autoimmune disease [1]. The prominent T cell dysfunction has molecular underpinnings in SLE [2,3]. The pathogenesis of systemic lupus erythematosus is complex and involves numerous immune cells and cytokines; accordingly, it remains unclear [4].

Recent studies have found that oxidative stress not only impacts the immune functions but also is related with the pathogenesis of SLE, where oxidative stress upregulated immune genes, and autoreaction by DNA demethylation conferred the risk of SLE [5,6]. In order to observe the influence of oxidative stress on DNA demethylation in the pathogenesis of SLE, we injected H_2_O_2_ treated CD4 + T cells into the tail vein of mice to establish a mouse-lupus-like model as in our previous study [7]. We found that downregulated oxidative stress increased the level of *cd40l* gene demethylation, which was associated with the progression of SLE. As a transmembrane glycoprotein, CD40 ligand (CD40L) belongs to the superfamily of tumor necrosis factor (TNF) and binds CD40 on the surface of B-cells as well as macrophages, which is primarily expressed in resting and activated T cells and shows induced patterns in activated T cells [8,9]. It has also been shown that CD40-CD40L interaction is critical for both auto-antibody production and deposition of immune complexes in kidneys, leading to kidney damage [10,11]. Besides, as one of the short-chain dehydrogenase/reductase (SDR) family members, 3-hydroxy butyrate dehydrogenase 2 (BDH2) accelerates the rate-limiting step of intracellular iron homeostasis. Depletion of iron by inhibiting BDH2 expression makes intracellular iron abnormally accumulated, resulting in iron deficiency in mitochondria, suggesting that BDH2 exerts a crucial influence to iron homeostasis inside the cells [12,13]. Our previous study found that the BDH2 deficiency was related to the iron homeostasis disorders among CD4 + T cells, which further involved in DNA demethylation plus self-reactive T cells in SLE [14]. The process of cell proliferation is positively regulated by BDH2, and, as a tumor suppressor, BDH2 is down-regulated in hepatocellular carcinoma and participates in regulating cell apoptosis and autophagy [15]. BDH2 stimulates ROS-induced cell apoptosis and autophagy in gastric cancer through the promotion of Nrf2 ubiquitination [16]. It also affects cell differentiation, apoptosis, and leukemic transformation of the myelodysplastic syndrome [13]. However, there are few studies on the effect of BDH2 on SLE pathogenesis.

Therefore, based on our previous results, we hypothesized that the BDH2 deficiency aggravated the SLE by promoting CD40L demethylation. Our aim was to explore the role of BDH2 in oxidative damage-induced SLE and to achieve the new therapeutic target. We found that downregulation of BDH2 promotes the pathologic progression of SLE in mice, by increasing the demethylation level of CD40L in CD4 + T cells. This might imply the discovery of novel BDH2 functions in SLE, which we study herein.

## Materials and methods

### CD4 + T cell isolation, culture, and transfection experiments, and establishment of a systemic lupus erythematosus mouse model

Female BABL/c mice (12 weeks old, 18–22 g) spleens were mechanically minced, and spleen lymphocytes were separated by density gradient centrifugation. Mouse spleens were placed on the filter, added with 15 ml of phosphate buffered saline (PBS) solution (containing 2% fetal bovine serum), and cut into a cube about 1 mm^3^. Then, 5–10 ml of lymphocyte separation liquid (GE-Healthcare) was added to the culture dish, and the mouse spleen was gently crushed so that the dispersed lymphocytes penetrated the filter and entered the separation liquid. Immediately, the cells solution was covered with 200 μl of 1640 medium, centrifuged at 800 × g for 30 min. The uppermost lymphocyte layer was collected. The isolation of CD4 + T cells from T cells was performed with anti-CD4 magnetic beads (Miltenyi Biotec GmbH), according to manufacturer’s instruction.

CD40L-shRNA plasmid or BDH2-shRNA plasmid were separately transfected into CD4 + T cells, then normal or transfected CD4 + T cells were treated with 50 μM H_2_O_2_ (Sigma) for 72 h. Treated CD4 + T cells (Trypan blue-staining cells <1%) were washed, and the intravenous injection of cells (5 × 10^6^) into the tail vein of BALB/C mice (12 weeks old, 18–22 g) was administered every 2 weeks for a total of 7 injections. However, the control group received intravenous injection of the empty vector (transfection-negative control).

To confirm that the SLE mouse model was established, Serum anti-dsDNA antibody, urine protein level, and hematoxylin and eosin-stained kidneys were used. Six mice for each group and three independent repeats for each experiment. About 1 ml of blood was collected from the eyeball. Half of the blood was used for flow cytometry, and serum was separated from the left part of the blood for the ELISA test. Urine samples were obtained every 5 weeks for urine protein examination.

## Real-time PCR assay

CD4 + T cells were initially isolated from spleens of SLE or control mice. Then, total RNA from CD4 + T cells was extracted using the Trizol reagent (Thermo Fisher Scientific). The reverse transcription from total RNA to cDNA was conducted through PrimeScript® RT reagent Kit (Takara, Japan), complying with the instructions provided by the manufacturer. In addition, the SYBR Premix Ex Taq II (Tli RnaseHPlus) (Takara, Tokyo, Japan) was adopted for all polymerase-chain reactions (PCR) to examine the mRNA levels of CD40L and BDH2. An initial denaturation step (95°C for 10 min) was carried out prior to PCR amplification, with over 40 cycles of denaturation for 15 s at 95°C as well as annealing and elongation for 1 min at 60°C. Besides, the specificity of the amplification reaction was determined by melting curve analysis. The comparative cycle threshold 2^−ΔCT^ method evaluated the relative expression of target genes, which were normalized to GAPDH. The primers are presented in (Table 1).

**Table 1.** Mouse primers for real-time PCR

Primers	Sequencing (5ʹ→3ʹ)
CD40L-F	CCTTGCTGAACTGTGAGGAGA
CD40L-R	CTTCGCTTACAACGTGTGCT
BDH2-F	CGACTGGACGGCAAAGTTATT
BDH2-R	CCTGGAGTTTGGACTCGTTGA
GAPDH -F	AGGTCGGTGTGAACGGATTTG
GAPDH -R	TGTAGACCATGTAGTTGAGGTCA

## Western blot assay

The total protein from CD4 + T cells was isolated from the spleens of the SLE or control mice as the mice model part. Protein quantification was done using the BCA quantification kit (Beyotime, China). 5 × loading buffer mixed with protein solution (Volume 4:1) boiled for 10 min for denaturation. 20 μg total protein was loaded in 10% SDS-PAGE. Proteins were transferred to PVDF (0.45 μm, Merck Millipore) membrane at constant voltage 100 V. Primary polyclonal anti-CD40L (1:5000) (ab52750, Abcam, USA), anti-BDH2 (0.4 µg/ml) (ab254710, Abcam, USA) as well as anti-GAPDH (1:5000) (60004-1-Ig, Proteintech, USA) antibodies were used to detect protein expression. Proteins were visualized with an ECL-chemiluminescent kit (Beyotime, China). The intensity of the band was measured by ImageQuant™ LAS 4000 mini (GE-Healthcare).

## Enzyme linked immunosorbent assay

Blood serum samples were obtained from mice of all groups and a dsDNA detection kit (Yansheng, China) was used to detect mouse Serum anti-dsDNA antibody in samples. Serum 10 μL was added into 40 μL diluted solution mixed with 100 μL. Natural DNA antibody (dsDNA) used as capture antigen and to coat the microplate making solid-phase antigen according to manufacturer’s instruction. Besides, an antigen-antibody enzyme-labeled antibody complex was formed with the combination of dsDNA and HRP labeled detection antibody. After thoroughly washing, the addition of tetramethylbenzidine (TMB) substrate solution was performed for color development. TMB turns blue with the catalysis of the HPR enzyme but turns yellow when reacting with the acid. The color was positively correlated with the amount of mouse Serum anti-dsDNA antibodies in the samples. A standard curve set up by a reference standard from the kit was used to calculate the concentration of anti-dsDNA.

Blood serum samples obtained from mice (1 week after the final cell injection) of all groups were used to detect the levels of IFN-γ, IL-2, IL-4 as well as IL-10 through enzyme-linked immunosorbent assay (ELISA) examination kits. The measurement of serum levels of cytokines was carried out through the double antibody sandwich method, according to the manufacturer’s instruction. According to the absorbance of standard and sample in OD450, the concentration of the target protein in the sample was calculated.

## Urinary protein levels were detected by Coomassie Brilliant Blue

Urine obtained from mice of all groups was used for urinary protein examination. Coomassie brilliant blue was adopted for the measurement of the average concentration of urinary protein during 24 h. We monitored the urinary protein of SLE mice at 5, 10, and 15 weeks after model establishment. Coomassie's brilliant blue G-250 dye (Solabio, China) combines with proteins in an acidic solution. The appropriate volume of urine was used to get the measured value within the linear range of the standard curve. According to the measured A595 nm value, the amount equivalent to the standard protein on the standard curve was determined, and then the protein concentration (mg/mL) of the unknown sample calculated.

## Hematoxylin and eosin staining

Kidney samples from mice were collected 14 weeks after the first injection, 4-μm sections of paraffin-embedded biopsies were cut and used for HE staining. HE staining revealed pathological changes in kidney tissue. The nucleus of the sample was dyed bright blue with hematoxylin. The cytoplasm was stained pink to crimson by eosin. After xylene dewaxing, ethanol hydration, hematoxylin and eosin staining, ethanol differentiation, and xylene transparent, the sections of kidney tissue were sealed with neutral gum before they were observed and photographed under a microscope (Samsung, Korea).

## Flow cytometry assay

Th1/2 cell subsets were detected by flow cytometry. After being isolated from PBMCs of SLE or control mice, CD4 + T cells were suspended at 1 × 10^7^ cells/mL in 100 μL cell staining buffer containing 10 μL FITC anti-Mouse CD4 (100405, Biolegend)/FITC anti-Mouse CD4 ISO (400605, Biolegend), and incubation was performed under dark conditions at room temperature for 20 min. The cells were fixed with a fixation buffer (420801, Biolegend) at room temperature for 20 min in the dark. The cells were then incubated with antibodies APC anti-mouse IFN-γ (505810, Biolegend), APC FN-γ ISO (400411, Biolegend), PE anti-Mouse IL-4 (504104, Biolegend), PE IL-4 ISO (400407, Biolegend) in the dark for 20 min at room temperature. Following washing with the buffer, the detection of cells was performed by flow cytometry (Beckman Coulter).

Reactive oxygen species (ROS) among CD4 + T cells were also detected through flow cytometry (Beckman Coulter). A detection kit based on the fluorescence probe DCFH-DA was adopted to determine the ROS generation. CD4 + T cells were harvested from mice spleens; besides, the cell concentration was regulated and reached 1 × 10^6^ cells/mL. Subsequently, cells were re-suspended and a treatment-containing ROS assay medium was added to the cell suspension. Cells were protected from light and incubated for 15 min at 37°C after which fluorescence activated cell sorting (FACS) analysis was carried out to study intracellular ROS.

## Methylation specific polymerase chain reaction

The methylation status of the *cd40l* DNA promoter region was evaluated using a methylation-specific polymerase-chain reaction (MSP). The concentration of CD4 + T cells purified from the spleen of control mice in each group was about 5 × 10^7^ cells and transfected with RNA interference (RNAi) plasmids of CD40L and/or BDH2. Genomic DNA was extracted (TIANGEN, China), and DNA methylation was determined after sodium bisulfite modification of genomic DNA. Methylation status of the *cd40l* promoter was analyzed using methylation and un-methylation primers (Table 2). After an initial denaturation step (5 min at 95°C), PCR amplification was performed over 35 cycles of denaturation for 20 s at 94°C, annealing for 30 s at 60°C as well as elongation for 20 s at 72°C, with a final extension of 72°C for 5 min before holding at 4°C. After the reaction, the amplified product (10 μl) was tested by agarose gel electrophoresis (1.5%).

**Table 2.** Primers for MSP of CD40L promoter

Primer type	Primer name	Sequencing (5ʹ→3ʹ)
Methylate	MSP-M-CD40L-Forward	TTTTGTTTGATTTTTTAAATATCGTT
	MSP-M- CD40L-Reverse	CTAATTTCATATCCAATATACCGCC
Un-methylate	MSP-U- CD40L-Forward	TTTTGTTTGATTTTTTAAATATTGT
	MSP-U- CD40L-Reverse	CTAATTTCATATCCAATATACCACC

## Cell proliferation assay

To evaluate the effect of *cd40l* or *bdh2* gene silencing on CD4 + T cell proliferation, CCK8 kit (Beyotime, China) was used. Cells were seeded at a cell confluence of 60–80% into the 6-well plates, and RNA interference (RNAi) plasmids of CD40L and BDH2 were transfected using Lipofectamine 3000 (Invitrogen, USA). In addition, after 6 h of transfection, the medium was abandoned for the complete culture medium, followed by 24 h of incubation. After being digested by pancreatic enzymes without EDTA, the cells were seeded at a density of 8 × 10^3^ cells per well in 96-well culture plates. The optical density (OD) values at 450 nm were measured at 24 h, 48 h, 72 h, and 96 h using a microplate reader (BioTek microplate reader).

## Detection of malondialdehyde level

Malondialdehyde (MDA) was measured using the thiobarbituric acid assay by lipid peroxidation MDA assay kit (Beyotime, China). CD4 + T cells (1 × 10^6^ cells/mL) were harvested with RIPA lysis buffer. After 10-min of centrifugation at 12,000 g, the supernatant was collected. Samples were heated at 100°C for 15 min, placed in a water bath, and cooled to room temperature. Two hundred microliters of the collected supernatants were added to a well of a 96-well plate, the absorbance of the supernatant was measured by spectrophotometer at 532 nm, and sample concentration was then calculated based on OD values.

## Statistical analysis

Statistical analysis was performed using the software of GraphPad Prism (version 8.0). The data are described with the mean ± standard deviation (SD). The experimental data were analyzed through one-way analysis of variance (ANOVA) or Tukey’s post hoc test. The value of P < 0.05 was recognized to be with statistical significance.

## Results

Lots of studies have reported on the implications of the CD40-CD40 ligand (CD40L) signaling pathway in SLE. This study explores the effect of BDH2 and CD40L in oxidative damage-induced SLE. Compared with SLE model mice, the levels of serum anti-dsDNA antibody and urinary protein in the CD40L knock-down group were significantly decreased by alleviated immune complex glomerulonephritis caused by CD4 + T cells treated with H_2_O_2_ in syngeneic mice. The levels of IFN-γ, as well as IL-2, were decreased accompanying with levels of IL-4 as well as the IL-10 upregulated. Inhibited BDH2 expression among CD4 + T cells promoted the CD40L’s demethylation, while it inhibited cell proliferation and elevated oxidative stress through increased expression of CD40L, and thus, promoted the progress of SLE.

## Identification of the role of CD40L in a systemic lupus erythematosus mouse model

**Figure 1.**
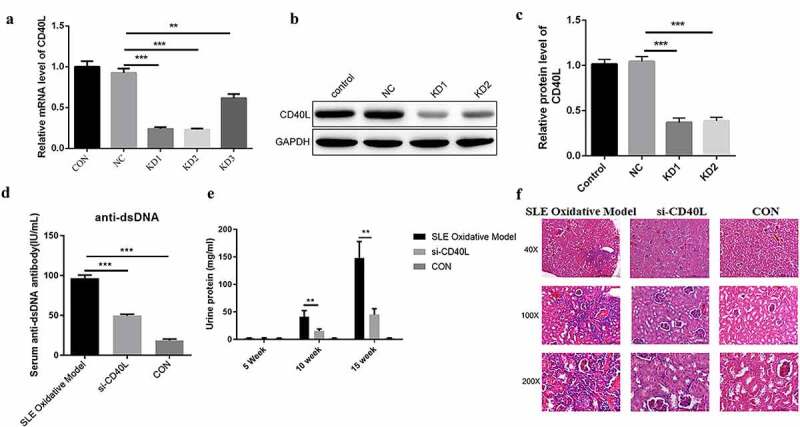
Identification of the role of CD40L in systemic lupus erythematosus mouse model (a) Quantitative PCR detects the CD40L mRNA level. Three shRNAs were designed targeting CD40L which were LV-CD40L-shRNA1 (KD-1), LV-CD40L-shRNA2 (KD-2), LV-CD40L-shRNA3 (KD-3). As showed in this part the knock down percentage of KD1 and KD2 was more than 50% and they were selected for the following experiment. ‘NC’ is the group transfected with lentivirus vector GV493. KD1, KD2, KD3 were GV493 insert with three shRNAs targeting CD40L. (b & c) Western blot detected the CD40L protein level. (d) Serum anti-dsDNA antibody level. (e) Urinary protein in mice was examined and CD40L alleviated the pathological changes in mouse kidneys. (f) HE staining detected the pathological changes in mouse kidneys. In (D)-(F) SLE oxidative model group, mice were injected with CD4 + T cells treated by H_2_O_2_; si-CD40L group, mice were injected with CD4 + T cells transfected with LV-CD40L-shRNA1 (KD-1). Control group, the negative control of transfection. Each group contained 6 mice and three independent repeats for each experiment. One-way ANOVA was used for statistical analysis. *P < 0.05, **P < 0.01, ***P < 0.001.

We first injected H_2_O_2_ treated CD4 + T cells into the tail vein of mice to establish a mouse lupus mouse model, as we did before [7]. In order to identify the effect of CD40L on SLE, CD4 + T cells were firstly infected with CD40L shRNA lentivirus plus treated with H_2_O_2_, followed by the intravenous injection into the tail vein of mice. It was found that we successfully interfered with the expression of CD40L in SLE model mice (Figure 1(a-c)). We next determined whether CD40L affected the pathogenesis of the SLE. By detecting the level of serum anti-dsDNA antibody and urinary protein in mice, and observing the pathological changes in SLE mouse kidneys, we further clarified that interfering with the expression of CD40L in SLE mice significantly reduced the level of serum anti-dsDNA antibody (Figure 1(d)) and urinary protein in mice (Figure 1(e)). At the same time, it alleviated the pathological changes in mouse kidneys (Figure 1(f)). Results presented above showed that the pathological process of SLE mice was alleviated by successfully interfering with the level of CD40L in SLE mice.

## CD40L decreased the proportion of Th1/Th2 cells in systemic lupus erythematosus mice

**Figure 2.**
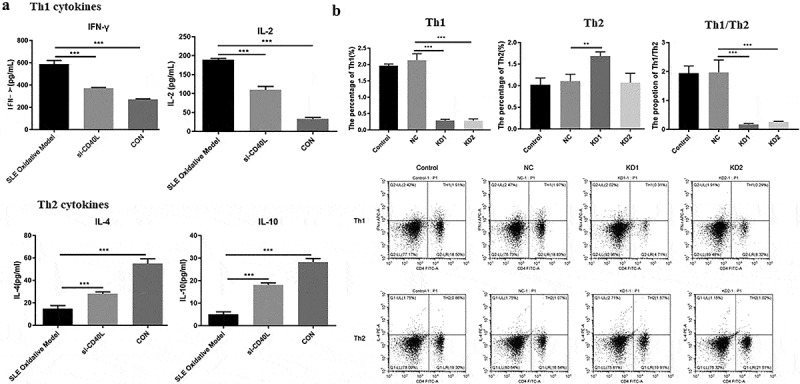
CD40L decreased the proportion of Th1/Th2 cells in systemic lupus erythematosus mice. (a) Cytokine level of IFN-γ, IL-2, IL-4 and IL-10 (p < 0.01). (b) Flow cytometry results Th1/Th2 cells. KD1 group, CD4 + T cells transfected with LV-CD40L-shRNA1 (KD-1); KD2 group, cells were treated with LV-CD40L-shRNA2 (KD-2). SLE oxidative model group, mice were injected with CD4 + T cells treated by H_2_O_2_, si-CD40L group, mice were injected with CD4 + T cells transfected with LV-CD40L-shRNA1 (KD-1). Control group, the negative control of transfection. Each group contained 6 mice and three independent repeats for each experiment. One-way ANOVA was used for statistical analysis. *P < 0.05, **P < 0.01, ***P < 0.001.

In order to further explore the regulatory role of CD40L in SLE, we first examined cytokine levels, such as IFN-γ, IL-2, IL-4, as well as IL-10, which were related to Th cell differentiation. Results showed that interfering with the expression of CD40L in SLE mice significantly reduced the levels of IFN-γ and IL-2, while it downregulated the levels of IL-4 as well as IL-10 (Figure 2(a)). The results of flow cytometry showed significantly decreased Th1 cells but increased Th2 cells in SLE mice, with the Th1/Th2 ratio also significantly decreased (Figure 2(b)). The above-described results suggest that interfering with the expression of CD40L in SLE mice suppresses inflammatory reactions, and it is well known that inflammatory cytokines have been demonstrated to contribute to SLE pathogenesis.

## BDH2 regulates the methylation of CD40L

**Figure 3.**
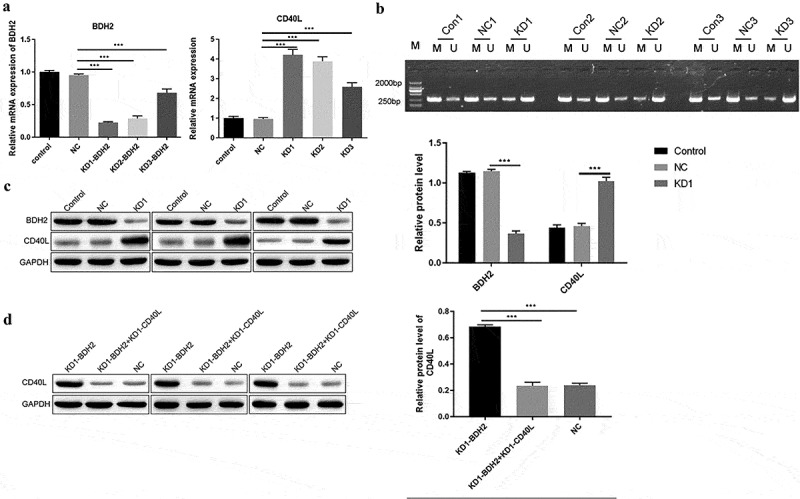
BDH2 upregulates the protein level of CD40L by inhibiting methylation. (a) Real-time PCR detected the expression of BDH2 and CD40L. Three shRNAs were designed targeting BDH2. They were LV-BDH2-shRNA1 (KD-1), LV-BDH2-shRNA2 (KD-2), LV-BDH2-shRNA3 (KD-3). As showed in this part the knock down percentage of KD1 was about 80%. And it was selected for the following experiment. ‘NC’ group, cells were transfected with lentivirus vector GV248. (b) Results of methylation specific PCR showed that the decrease of BDH2 resulted in the increase of demethylation level of CD40L. (C&D) Western blots showed that BDH2 upregulates the CD40L protein level by inhibiting CD40L methylation. Three independent repeats for each experiment and One-way ANOVA used for statistical analysis. *P < 0.05, **P < 0.01, ***P < 0.001.

We further investigate the effect of BDH2 on the pathogenesis of the SLE by regulating CD40L. The combined mRNA expressions of BDH2 and CD40L were detected among CD4 + T cells after *bdh2* interference. After interfering with the expression of BDH2, the mRNA level of BDH2 was indeed decreased, opposite with that of CD40L (Figure 3(a)); Then, we observed the effect of interference with BDH2 on the methylation level of the CD40L promoter region, and results showed that the decrease in BDH2 led to the increased demethylation of CD40L (Figure 3(b)), thereby increasing CD40L protein level (Figure 3(c)). The rescue experiment indicated CD40L protein level decreased to a level similar to that of NC group when the cells were transfected with both sh-BDH2 and sh-CD40L (Figure 3(d)). The above research results showed that knocking-down of BDH2 upregulated CD40L protein level by inhibiting CD40L methylation, which may affect the pathological process of SLE.

## BDH2 promotes cell proliferation, decrease oxidative stress, aggravated the pathological process by inhibiting the expression of CD40L

**Figure 4.**
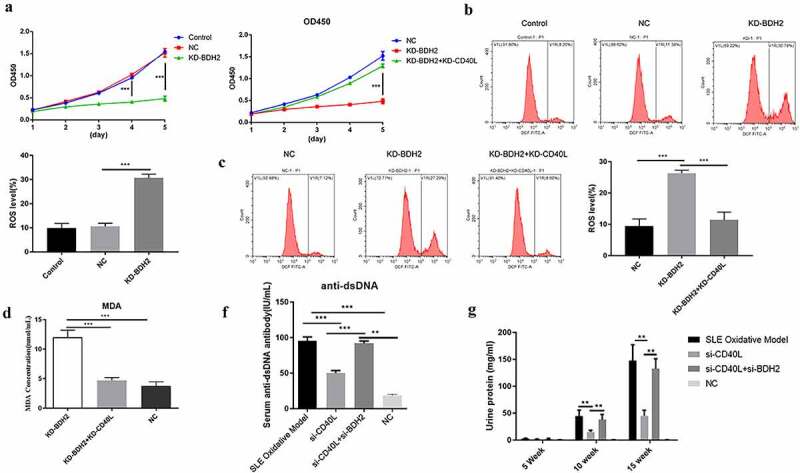
Effect of BDH2 on CD4 + T cell function and aggravation of the pathological process of systemic lupus erythematosus (a) Proliferation effects when BDH2 was solely or simultaneously interfered with CD40L interference among CD4 + T cells. (b&c) Results of flow cytometry showed that interfering with BDH2 expression among CD4 + T cells promoted level of ROS, while CD40L was simultaneously interfered with BDH2 interference among CD4 + T cells, the level of ROS was inhibited. (d) Data showed that interfering with BDH2’s expression promoted the levels of MDA, while after the interference with the combined expressions of both BDH2 and CD40L, cells fell to baseline levels. (f) Serum anti-dsDNA antibody level. (g) Urinary protein in mice was examined. ‘NC’ group was transfected with lentivirus vector GV248. ‘KD-BDH2’ group was transfected with LV-BDH2-shRNA1 (KD-1), ‘KD-BDH2-KD-CD40L’ group was transfected with both LV-BDH2-shRNA1 and LV-CD40L-shRNA1. Three independent repeats for each experiment and One-way ANOVA used for statistical analysis. *P < 0.05, **P < 0.01, ***P < 0.001.

Based on the above findings regarding either *in vitro* or *in vivo*, we studied BDH2’s influence on CD4 + T cell’s function, finding the inhibited cell proliferation among CD4 + T cells after interfering with BDH2’s expression, in contrast to control and negative control groups (left curve of Figure 4(a)); while CD40L was simultaneously silenced in CD4 + T cells where BDH2 was target of interference, the cell proliferation ability was significantly improved (right curve of Figure 4(a)). Interfering with BDH2’s expression among CD4 + T cells promoted the level of ROS (Figure 4(b)). However, when the CD40L was simultaneously silenced by silenced BDH2 among CD4 + T cells, ROS level decreased (Figure 4(c)). These results showed that BDH2 increased ROS level by promoting the expression of CD40L. The results of another oxidative stress test showed that interfering with BDH2’s expression promoted the levels of MDA. However, when interfering with the combined expressions of BDH2 and CD40L, cells fell to baseline levels (Figure 4(d)). By detecting the level of serum anti-dsDNA antibody and urinary protein in mice, we further clarified that interfering with the expression of CD40L and BDH2 in systemic lupus erythematosus mice simultaneously recovered the level of serum anti-dsDNA antibody (Figure 4(f)) and urinary protein in mice (Figure 4(g)) comparing with mice only interfering with CD40L. The above results showed that BDH2 inhibited cells proliferation and promoted oxidative stress by promoting the expression of CD40L so as to aggravate the pathological process of SLE.

## Discussion

SLE is an autoimmune inflammatory disease (AID) occurring in connective tissues, with the involvement of multiple organs in young women [17]. The etiology of the disease has not been confirmed yet but based on the results of the studies, the abnormalities caused by genetic, infectious, immunizing factors, as well as some environmental factors, were all involved in SLE pathogenesis [18,19]. In other words, genetic and shared environmental risk factors were related to the reduction of T lymphocytes, the functional inhibition of T suppressor cell, the superfluous proliferation of B cells, the massively produced auto-antibodies, and the combination with the corresponding auto-antigens inside the body to form immune complexes, which are subsequently deposited in the skin, joints, small blood vessels, glomerulus, as well as other areas [20–22]. CD40L is one of the tumor necrosis factor superfamily; besides, it is also an important molecule involved in the process of autoimmune response. Therefore, its antibody has the potential for targeted therapy in autoimmune diseases [23,24]. T cell activation is very important in the pathogenesis of SLE [25]. CD40/CD40L was crucial in T cell activation, proliferation, and antibody production; CD40/CD40L signal activation can produce different effects, which could promote cell proliferation and differentiation, but also inhibit cell proliferation and apoptosis [26,27]. We found that Th cells acted on B cells through CD40/CD40L cross-linking to induce cell activation, proliferation, and antibody production [28,29]. T cells in SLE patients expressed high levels of CD40L and the expression period was prolonged [30,31]. Some research found a high level of soluble CD40L in serum of SLE patients, and its titer was related to the severity of the disease [32]. The level of CD40/40 L in renal tissue of patients with lupus nephritis significantly increased. CD40/CD40L co-stimulatory molecular pathway controls the production of antigen-dependent antibodies and participates in the occurrence and development of SLE. Therefore, blocking the interaction between CD40 and CD40L prevents T cell activation and B cell immune response, as well as reduces SLE symptoms. The level of dsDNA antibody was decreased significantly when PBMC in patients with active SLE was incubated with CD40L monoclonal antibody [33]. The present study indicates that blocking CD40L might delay the progression of SLE. Our data suggested that the inhibited CD40L expression among CD4 + T cells reduced dsDNA antibody concentration in serum and urinary protein in urine of SLE mice, while it alleviated lupus nephritis. Based on the good effect of anti-CD40L antibody in the treatment of SLE mice, anti-CD40L monoclonal antibody had been applied to the clinical treatment of human SLE.

Given the important role of the CD40L in SLE pathogenesis, the study of the mechanism of regulation of CD40L expression was beneficial for understanding SLE pathogenesis, as well as developing more effective therapeutic drugs. At present, most studies focus on the development of CD40L monoclonal antibody, and little is known about the proteins that regulate CD40L expression. The study found that E4BP4 could inhibit CD40L expression by means of epigenetic modifications in CD40L promoter region, leading to the negative regulation of the SLE self-reactivity [34]. For SLE patients, the peroxisome-proliferator activated receptor (PPAR) -γ expression is increased. Meanwhile, the CD40/CD40L signaling pathway is repressed [35]. According to the results of our previous study, BDH2 was involved in regulating CD40L expression in SLE mice. Studies have found that BDH2-related diseases included α-methylacetoacetic aciduria, myelodysplastic syndrome, and gastric cancer [13,16]. The related pathways are involved in regulating lipid metabolism by PPAR-α plus ketone body metabolism. As the dehydrogenase, which mediated 2, 5-dihydroxybenzoic acid’s formation, BDH2 exerted a critical influence on iron assimilation and homeostasis as well as the susceptibility to infectious diseases based on an assimilable source of iron exploited by pathogenic bacteria; besides, it also plays a role in a 3-hydroxybutyrate dehydrogenase [36]. BDH2 could be upregulated by decreased levels of iron, however, down-regulated by elevated levels of iron. Studies have found that as an anti-apoptosis factor in humans, BDH2 is a novel factor for the poor prognosis of *de novo* cytogenetically normal acute myeloid leukemia (AML) [37].

## Conclusion

In conclusion, BDH2 alleviated the pathological process of SLE. Furthermore, for SLE mice, by simultaneously interfering with the combined expressions of BDH2 and CD40L among CD4 + T cells, BDH2 could delay SLE progression by promoting methylation, thereby inhibiting the expression of CD40L. Downregulated BDH2 promoted the demethylation of CD40L and the increase in CD40L expression, which promoted the pathological process of the SLE. This is a novel finding with relevance for SLE pathogenesis, which might help in the treatment of SLE.

## Data Availability

The data used to support the findings of this study ara included within the article, further inquiries can be directed to the corresponding author.

## Highlights

1. A conformational mechanism for CD40L’s exacerbation in oxidative SLE is proposed.
2. The mechanism is related to BDH2’s promotion in the CD40L’s demethylation.
3. Demethylation of CD40L elevated oxidative stress and promotes the process of SLE.
